# Depressive symptoms and clustering of risk behaviours among adolescents and young adults attending vocational education: a cross-sectional study

**DOI:** 10.1186/s12889-015-1692-7

**Published:** 2015-04-18

**Authors:** Rienke Bannink, Suzanne Broeren, Jurriën Heydelberg, Els van’t Klooster, Hein Raat

**Affiliations:** Department of Public Health, Erasmus University Medical Center Rotterdam, P.O. Box 2040, 3000 CA Rotterdam, the Netherlands; Municipality of Rotterdam, Librijesteeg 4, 3000 KS Rotterdam, the Netherlands; Public Health Care for Youth, Westblaak 171, 3012 KJ Rotterdam, the Netherlands

**Keywords:** *Risk behaviours*, *Depression*, *Clustering*, *Substance use*, *Adolescents*, *Young adults*

## Abstract

**Background:**

Depressive symptoms and risk behaviours often do not occur in isolation among adolescents and young adults. In order to improve intervention programmes, more research is needed to elucidate the clustering of risk behaviours, the association with depressive symptoms, and demographic variables. Therefore, this study examined the clustering of risk behaviours, the association with depressive symptoms, and demographic variables among adolescents and young adults in vocational education. Furthermore, the prevalence of depressive symptoms and risk behaviours was examined.

**Methods:**

This study included 584 students (mean age 18.3 years) attending vocational education in the Netherlands. Depressive symptoms and risk behaviours (binge drinking, cannabis use, smoking, delinquency and incurring debts) were assessed with self-report questionnaires. Truancy was monitored via the school registration system. Principal Components Analysis (PCA) was conducted to assess the factor structure of the risk behaviours (i.e. clustering). Linear regression analyses with a bootstrapping method were performed to assess the associations.

**Results:**

Binge drinking was reported by 50.5% and cannabis use by 14.2% of the students (both in the past 4 weeks), whereas 37.7% reported currently being a smoker. More than 10% reported having been questioned at a police station in the past year. Furthermore, 82.2% had been truanting in the first two months of education, 21.0% reported having debts and 29.2% reported clinically-relevant depressive symptoms. The PCA indicated two clusters. The ‘substance use’ cluster consisted of the risk behaviours: binge drinking, cannabis use and smoking. The ‘problem behaviours’ cluster consisted of the risk behaviours: delinquency, truancy and incurring debts. Both clusters were associated with depressive symptoms. Various demographic variables were associated with both clusters.

**Conclusions:**

Risk behaviours formed two clusters, both of which were associated with depressive symptoms. These findings underscore the importance of screening adolescents and young adults at lower educational levels for multiple risk behaviours and depressive symptoms and of focusing on multiple risk behaviours in interventions simultaneously.

**Electronic supplementary material:**

The online version of this article (doi:10.1186/s12889-015-1692-7) contains supplementary material, which is available to authorized users.

## Background

A high percentage of adolescents and young adults suffer from depressive symptoms and display many risk behaviours such as substance use, delinquency, truancy and making purchases they can not afford, which are acquired during adolescence [1]. By increasing the risk of developing major diseases such as cancer, cardiovascular disease, and psychiatric and psychosocial disorders, depressive symptoms and risk behaviours contribute to the public health burden [2,3]. Furthermore, depressive symptoms and risk behaviours often persist into adulthood, thereby affecting not only current health but also health later in life [2,4]. Furthermore, adolescents and young adults experiencing depressive symptoms or displaying risk behaviours are at increased risk of school dropout [5-9]. This phenomenon seems especially true for students attending vocational education. For example, in the Netherlands, 75% of school dropouts occur in vocational education [10]. As the senior level of the vocational track in Dutch secondary education, vocational education provides specialised vocational training to students aged 15 years and older.

According to studies, dropping out of school results in substantially lower earnings over the course of life [11], considerably more dependence on public assistance [12], and a substantially higher likelihood of involvement in crime and incarceration [13]. Since dropout often experience problems and exhibit risk behaviours earlier on, it is essential to gain a greater understanding of these problems and behaviours in order to prevent dropout and the associated problems later in life. However, little is known about the prevalence of risk behaviours among students in vocational education, especially delinquency, truancy and incurring debts.

According to Jessor’s problem behaviour theory, risk behaviours (e.g. drinking alcohol, delinquent behaviour) tend to co-occur in youth [14]. In previous research it was also shown, for example, that risk behaviours related to substance use (i.e. alcohol use, drug use and cigarette smoking) often cluster in adolescents [15,16]. However, most studies on health behavioural clustering have focused on a relatively small range of health behaviours and fail to examine the clustering of a wide range of risk behaviours such as delinquency, truancy and incurring debts [15,17]. Investigating the clustering of health risk behaviours is important because individuals with multiple health risk behaviours are at the greatest risk of developing chronic diseases and disabilities [15,18-20]. Understanding the prevalence of these behavioural clusters may inform health improvement planning efforts [20]. In addition, if risk behaviours cluster, prevention programmes aimed at changing clusters of risk behaviours, rather than separate risk behaviours, could lessen the burden on public health services. Therefore, the development of a prevention strategy to target multiple health risk behaviours simultaneously could be useful when behaviourscluster and have an underlying basis and similar predictors [17]. Although many public health intervention strategies still focus on behaviours in isolation, research has shown that risk behaviours related to substance use are responsive to such an integrated approach [21]. Furthermore, the World Health Organization (WHO) has adopted a holistic approach to health that emphasises prevention by tackling combinations of risk factors [19].

Additionally, previous research has suggested an association between depressive symptoms and substance use [22-25]. However, knowledge about relationships between depressive symptoms and other clusters of behaviours including delinquency, truancy and incurring debts is scarce. It is important to examine the association between different clusters of behaviours and depressive symptoms to further improve intervention programmes, and especially to improve the early identification of those at risk of multiple risk behaviours and/or depressive symptoms. Furthermore, to further improve intervention programmes, it is also important to examine if demographic characteristics can be used to identify adolescents and young adults at risk. Although research suggests that demographics can be used to identify adolescents displaying single risk behaviours or experiencing depressive symptoms, research on whether demographics can be used to identify adolescents and young adults at risk of multiple, clustered risk behaviours, especially clusters including delinquency, truancy and debts, is rare [26,27].

Overall, the purpose of this study was to examine the prevalence of depressive symptoms and risk behaviours (binge drinking, cannabis use, smoking, truancy, delinquency and incurring debts) among adolescents and young adults in vocational education. It also examined the clustering of risk behavioursand the association between the clusters and depressive symptoms and between the clusters and demographic variables (i.e. gender, ethnicity, age, and being a parent).

## Methods

### Participants and recruitment

This study used data obtained upon enrolment (pre-test measure) in the Your Health study, a cluster randomised controlled trial. The pre-test measure that was used in this study was conducted in 2012 and before randomisation had taken place. The intervention study is described in detail elsewhere [28]. A total of 44 first-year classes of students in vocational education in the Rotterdam region of the Netherlands participated. There are four levels of vocational education in the Netherlands. In this study, students from the two lowest levels of vocational education (the easiest levels) participated. The two lowest levels of vocational education last one to three years and focus on basic practical tasks. Only students from the two lowest levels were included, since studies have shown that the prevalence of risk behaviours among these students is high and school dropout rates are the highest of any group [6,7,29,30].

A few weeks prior to the start of the study, all students and parents received information about the study. If parents did not want their child to participate, they could object to the participation of their child (until adolescent age 18 years). During a classroom session, students who were present in class, were asked written consent before they completed a questionnaire. Written consent was provided by 70.4% (N = 584) of students. The main reason for non-participation was absence at time of the assessment.

The Medical Ethical Committee of Erasmus MC has declared that the Medical Research Involving Human Subjects Act (also known by its Dutch abbreviation WMO) does not apply to this research proposal. The Medical Ethical Committee had no objection against the execution of this research proposal (MEC-2012-367).

### Measurements

At school, during one classroom session (+/− 45 min), students completed a self-report questionnaire. Risk behaviours were assessed by items based on existing instruments previously developed by Municipal Public Health Services and health institutes in the Netherlands [31]. Truancy was monitored via the school registration system.

#### Demographics

Demographic characteristics included age, gender, country of birth of the students and both parents, and whether or not the student already was a parent him/herself. Ethnicity was classified as Dutch or non-Dutch, in accordance with the definitions of Statistics Netherlands [32].

#### Depressive symptoms

Symptoms of depression were assessed by the Center for Epidemiologic Studies Depression Scale (CES-D) [33]. The CES-D consists of 20 items. The frequency of symptoms is rated on a 4-point scale. Items scores are summed (range from 0–60), with higher scores indicating higher levels of depressive symptoms (current study α = 0.89). A cut-off point of 16 is used to indicate clinically significant depressive symptoms. This cut-off score corresponds with the 80th percentile in community samples [34]. This cut-off point was used to determine the percentage of students with clinically significant depressive symptoms. For the remaining analyses, the (continuous) total CES-D score was used.

#### Risk behaviours

Binge drinking was defined as 5 or more alcoholic drinks consumed on one occasion, a commonly used definition [35]. Students in this study were asked to report the number of times they consumed 5 or more drinks on one occasion in the past 4 weeks (never–9 or more times). Cannabis use was assessed by the number of times the student had used cannabis over the past 4 weeks (never–20 or more times). Cigarette smoking was assessed by how often the student smoked at time of assessment (not–every day). Delinquency was assessed by the item: “In the past 12 months, have you been questioned at a police station, because you were accused of doing something that was not permitted?” (never–6 or more times). Debts were assessed by the items: 1) do you have any debts (yes/no/don’t know), and 2) approximately how high is the sum of all your debts (less than 50 euro–more than 2500 euro). School administrators registered every hour of impermissible absence (i.e. absence without notification or valid reason) in the school’s registration system. Truancy was defined as the number of hours students were absent impermissible over the two months at the start of the study. Truancy data is not available for part of the students (12.5%; n = 73) due to school dropout, other reasons for leaving the school (e.g. switching schools), and failure to match the data.

Chi-square tests and *t-*tests were conducted to compare students from whom truancy information was or was not available. Group differences were found, with the students from whom no truancy information was available, more often being of non-Dutch ethnicity (*χ*^*2*^ = 5.48; *p* = .02), binge drinkers (*χ*^*2*^ = 7.37; *p* = .007), cannabis users (*χ*^*2*^ = 9.52; *p* = .002), cigarette smokers (*χ*^*2*^ = 4.64; *p* = .03), reporting delinquency (*χ*^*2*^ = 16.76; *p* < .001), incurring debts (*χ*^*2*^ = 4.60; *p* = .03), and having depressive symptoms (*t* = 3.23; *p* = .002).

For describing the prevalence of single risk behaviours ordinal scales were used. For other analyses purposes, risk behaviours were categorized as follows: binge drinking in the past 4 weeks (yes/no); cannabis use in the past 4 weeks (yes/no); current cigarette smoker (yes/no); delinquency in the past 12 months (yes/no); incurring debts (yes/no); truancy more than 2 hours in two months (yes/no).

### Statistical analyses

Statistical analyses were performed using SPSS version 21. Principal Components Analysis (PCA) with direct oblimin rotation was conducted to assess the factor structure of the risk behaviours (i.e. clustering of risk behaviours). The following criteria were used to determine the number of clusters: factors must have an eigenvalue of >1.00 and factor loadings must have an absolute value of >0.40. Furthermore, it was checked whether no substantial secondary factor loadings (i.e., >0.40) emerged [36]. Subsequently, factor scores according to cluster were computed for each student by adding up all of the risk behaviour score weights by their factor loading [37]. The factor scores were then used as separate variables in linear regression analyses. Linear regression analyses were performed to explore associations between: clustering of risk behaviours and depressive symptoms, demographics and depressive symptoms, and demographics and clustering of risk behaviours. For the linear regression analyses, a bootstrapping method was used [38]. This method deals with data that are skewed, as is often the case with data on depressive symptoms, and in this study. Additional logistic regression analyses treating depressive symptoms as binary outcome, instead of as continuous outcome, were also conducted (see Additional file 1 and Additional file 2). Any *P* values of < .05 were considered statistically significant.

## Results

### Students’ characteristics

The average age of the students in this study was 18.3 years (*SD* = 2.59). The majority (52.6%) of the students was under the age of 18, 43.0% was between 18 and 24 years old and 4.5% was 25 years or older. Of the students in this study, 38.9% was male and 10.6% was a parent (Table 1). The majority (62.1%) was of non-Dutch ethnicity.

**Table 1:** **Demographics, risk behaviours and depressive symptoms of the study population (N = 584)**

	**Total**
	**N = 584**
**Age in years**	
Mean **[** 2 **]**	18.3 (SD = 2.59, range 15–30)
	**%**
**Gender** **[** 1 **]**	38.9
Boys	
**Ethnicity** **[** 4 **]**	
Dutch	27.9
Surinamese	10.3
Antillean	15.7
Moroccan	6.4
Turkish	9.0
Cape Verdean	5.0
Other	25.7
**Being a parent** **[** 28 **]**	
Yes	10.6
**Binge drinking (past 4 weeks)** **[** 16 **]**	
Never	49.5
1 time	18.7
2 times	13.4
3 - 4 times	8.6
5 or more times	9.9
**Cannabis use (past 4 weeks)** **[** 8 **]**	
Never	85.8
1 – 4 times	4.5
5 or more times	9.7
**Cigarette smoking (currently)** **[** 11 **]**	
No	62.3
Yes, but not daily	8.0
Yes, daily	29.7
**Delinquency** **[** 6 **]**	
Questioned at a police station (last year)	11.1
**Truancy (past two months)** **[73]**	
Never	17.8
1 – 2 hours	15.9
3 – 10 hours	30.5
>10 hours	35.8
**Debts** **[** 46 **]**	
None	79.0
<500 euro	7.1
>500 euro	13.9
**Depressive symptoms** **[** 16 **]**	
CES-D score in the clinical range (score ≥ 16)^a^	29.2
CES-D score^b^, mean (SD)	12.5 (9.49)

*Note:* [missing data].

^**a**^ A cut-off point of 16 is used to indicate clinically significant depressive symptoms and corresponds to the 80^th^ percentile in community samples [34].

^**b**^ A higher score on the CES-D indicates higher levels of depression symptoms (range 0–60).

### Risk behaviours and depressive symptoms

Binge drinking was reported by 50.5% and cannabis use by 14.2% of the students (both in the past 4 weeks), whereas 37.7% reported currently being a smoker (Table 1). More than 10% reported having been questioned at a police station in the past year because they were accused of doing something that was not permitted. Furthermore, 82.2% had been truanting one or more hours in the past two months, 21.0% reported having debts and 29.2% reported clinically-relevant depressive symptoms

### Clustering of risk behaviours

The PCA yielded two factors with eigenvalues >1.00 with all factor loadings being >0.40 on one of the two factors, and low secondary factor loading (i.e., <0.40) (Table 2). The first factor consisted of three risk behaviours: binge drinking (*r* = 0.74), cannabis use (*r* = 0.74) and smoking (*r* = 0.73). This cluster was therefore named ‘substance use’. The second factor also consisted of three risk behaviours: delinquency (*r* = 0.50), truancy (*r* = 0.69) and incurring debts (*r* = 0.72). This cluster was named ‘problem behaviours’. There was a small correlation between the two clusters (*r* = 0.16, *p* = .001). Of the students, 33.2% reported the use of one substance, 18.1% the use of two substances, and 11.0% the use of three substances. Furthermore, 48.8% reported one problem behaviour, 19.3% two problem behaviours, and 2.3% three problem behaviours.

**Table 2:** **Factor structure and loadings of the risk behaviours** ^**a**^

**Risk behaviours**	**Loadings**	
	**Factor 1**	**Factor 2**
	**Substance use**	**Problem behaviours**
Binge drinking	0.74	−0.17
Cannabis use	0.74	0.06
Cigarette smoking	0.73	0.16
Delinquency	0.05	0.50
Truancy	−0.002	0.69
Debts	0.05	0.72
Eigenvalue	1.77	1.17
% Explained variance	29.55	19.53

^a^ Principal Components Analysis.

### Associations between clustering of risk behaviours and depressive symptoms

The substance use and problem behaviours clusters were significantly associated with depressive symptoms (Table 3). A higher score on the substance use cluster was associated with more depressive symptoms (B 1.61, 95% CI 0.49 – 2.55). A higher score on the problem behaviours cluster was also associated with more depressive symptoms (B 1.30, 95% CI 0.23 – 2.47). After adjusting for the other cluster, the beta coefficients remained significant (substance use: B 1.45, 95% CI 0.48-2.46; problem behaviour: B 1.04, 95% CI 0.04-2.16).

**Table 3:** **Associations between clusters of risk behaviours and depressive symptoms (N = 424)** ^**a**^

	**Depressive symptoms**		
	**Model 1a**	**Model1b**	**Model 2**
	**Beta coefficient (95% CI)**	**Beta coefficient (95% CI)**	**Beta coefficient (95% CI)**
Substance use	**1.61 (0.49 – 2.55)**		**1.45 (0.48 – 2.46)**
Problem behaviours		**1.30 (0.23– 2.47)**	**1.04 (0.04 – 2.16)**

*Note:* Bold numbers indicate significant results at *P* < .05.

^a^ Linear regression analyses using a bootstrapping method.

Model 1a is adjusted for age, gender, ethnicity, being a parent and substance use.

Model 1b is adjusted for age, gender, ethnicity, being a parent and problem behaviours.

Model 2 is adjusted for age, gender, ethnicity, being a parent, substance use and problem behaviours.

### Associations between demographics, clusters of risk behaviours and depressive symptoms

Age was associated with the problem behaviours cluster (Table 4). Older students reported more often problem behaviours (B 0.11, 95% CI 0.05-0.16). Girls reported more depressive symptoms than boys (B 3.56, 95% CI 2.18-5.15). Ethnicity was significantly associated with the cluster substance use and problem behaviours. Students of non-Dutch ethnicity less often reported using substances (B −0.51, 95% CI −0.72 to −0.29), but more often reported problem behaviours (B 0.31, 95% CI 0.11-0.53) compared to students of Dutch ethnicity. Separate exploratory analyses, in which each non-Dutch ethnicity was compared to Dutch ethnicity, yielded a similar pattern of results for each non-Dutch ethnicity. Finally, being a parent was associated with reporting more often problem behaviours (B 0.56, 95% CI 0.19-0.95).

**Table 4:** Associations between demographics, clusters of risk behaviours and depressive symptoms^a^

	**Substance use** ^**b**^	**Problem behaviours** ^**b**^	**Depressive symptoms** ^**b**^
	**Beta coefficient (95% CI)**	**Beta coefficient (95% CI)**	**Beta coefficient (95% CI)**
	**N = 432**	**N = 432**	**N = 534**
Age	0.04 (−0.02 – 0.09)	**0.11 (0.05 – 0.16)**	0.20 (−0.19 – 0.60)
Gender (ref. = boys)	−0.10 (−0.33 – 0.10)	−0.16 (−0.34 – 0.01)	**3.56 (2.18 – 5.15)**
Ethnicity (ref. = Dutch)	**−0.51 (−0.72 – -0.29)**	**0.31 (0.11 – 0.53)**	1.34 (−0.55 – 3.25)
Being a parent (ref. = No)	0.14 (−0.26 – 0.61)	**0.56 (0.19 – 0.95)**	−1.10 (−3.84 – 2.51)

*Note:* Bold numbers indicate significant results at *P* < .05.

^a^ Linear regression analyses using a bootstrapping method.

^b^ Age, gender, ethnicity and being a parent are included at the same time.

## Discussion

This study shows that risk behaviours and depressive symptoms are prevalent among adolescents and young adults attending vocational education. The results suggest that clustering of risk behaviours occurs. More specifically, the risk behaviours examined occured in two clusters: substance use (i.e. alcohol use, cannabis use and cigarette smoking) and problem behaviours (i.e. incurring debts, truancy and delinquency). Furthermore, both clusters of risk behaviours were associated with depressive symptoms. In addition, various demographic characteristics were associated with the clusters of risk behaviours and depressive symptoms.

Each of the individual risk behaviours was prevalent among the study population, with truancy having an especially high prevalence. That is, more than 4 out of 5 students had been truanting in the first two months of education. This is very worrying since truancy is a risk factor for school dropout, as are the other risk behaviours included in this study [5-9]. To the best of our knowledge, there have been no previous studies examining the prevalence of truancy (in hours) among students attending vocational education, as registered by a school registration system. Most often truancy is measured by self-report measures, which is a less objective measure than a school registration system.

The prevalence of cannabis use, cigarette smoking, depressive symptoms and incurring debts was high, though comparable with other studies among students attending vocational education [29,30,39]. However, binge drinking was more prevalent in our study (50.5%) compared to the study of Vogel et al. in which 33.2% of students attending vocational education reported having been binge drinking in the past 4 weeks [29]. This discrepancy may be due to differences in the level of education; the study by Vogel et al. included students from all four levels of vocational education, whereas our study only included students from the two lowest levels. Students at a lower education levels have a greater tendency to drink large amounts of alcohol compared to students at higher levels of education [40]. This difference is probably attributable to the fact that students at lower levels spend more time with their peers and are not supervised by their parents as much, both of which are associated with more drinking [27]. Studies examining the prevalence of delinquency among adolescents and young adults attending vocational education seem to be lacking and therefore more research is needed. This is especially true given that more than 10% of students in our study reported that they were questioned at a police station in the past year after being accused of breaking the law.

Two clusters of risk behaviours were identified (i.e. substance use and problem behaviours). The clustering of substance use-related risk behaviours (i.e. alcohol use, cannabis use, and cigarette smoking) was also found in other studies among adolescents in general [15,16,21], whereas prior research among students attending vocational education showed an association between binge drinking, cannabis use and cigarette smoking [29]. The clustering of the use of different substances has been explained by so-called gateway theories and by a shared determinant that increases the risk of using substances in general. Gateway theories state that the use of one substance leads to experimentation and use of other substances [41]. Alternatively, a shared determinant, such as a personality trait (e.g. novelty seeking) that makes it more likely a student will experiment with substances, or an environment in which students are exposed to substance use and/or abuse by the example of parents or friends, could increase students’ risk of multiple substance use [42].

The other cluster, problem behaviours, comprised the risk behaviours incurring debts, truancy and delinquency. Although previous research showed an association between incurring debts and delinquency [43], between delinquency and truancy [8,44], and between incurring debts and active participation at school among students [45], it appears that the clustering of these three has never before been investigated. The clustering of these risk behaviours may be explained by the Strain Theory, which posits that financial problems are a source of strain in young people [46]. If these youngsters are not capable of dealing with strain in a legal manner, the risk of committing a minor violation, e.g. truancy or substance use, and delinquency may increase. Although the use of substances by adolescents is considered illegal behaviour in some countries, in the Netherlands the use of substances by adolescents is legal. That is, until 2013 the purchase of alcohol and cigarettes was allowed for those 16 and over (starting in 2014 the age was raised to to 18), and the use of cannabis is allowed for those 18 and over.

The clustering of risk behaviours suggests that interventions should preferably focus on multiple risk behaviours simultaneously rather than on separate risk behaviours in order to lessen the burden on public health services [17,18]. Because multiple risk behaviours were relatively common in the study population, preventive interventions targeting students attending vocational education and focusing on multiple behaviours simultaneously could be especially beneficial. However, to date, most intervention programmes still take a single risk behaviour approach, instead of an integrated one [21]. The finding of separate clusters indicates that some combinations of risk behaviours, i.e. those which form clusters could potentially be responsive to an integrated prevention approach. Moreover, it is of interest to examine whether the risk behaviours included in certain clusters have a shared determinant, such as a personality trait (e.g. novelty seeking) or a specific family environment (e.g. an environment with a lot of violence). Although the present research only focused on risk behaviours, some of the most promising intervention programme approaches for reducing multiple risk behaviours simultaneously address multiple domains of risk and protective factors predictive of risk behaviour [21].

Furthermore, our study shows that both clusters of risk behaviours were associated with depressive symptoms. This observation supports findings by Clark et al. and Boys et al., which demonstrate that adolescents who engaged in more health risk behaviours (i.e. smoking, alcohol, and/or drug use) were at increased risk of depressive symptoms [23,24]. Therefore, if multiple risk behaviours are evident in adolescents and young adults, it could be useful to screen for and address depressive symptoms, whereas if depressive symptoms are evident it could be useful to screen for and address multiple risk behaviours. This approach may help to improve the early identification of those at risk of multiple risk behaviours and/or depressive symptoms.

Moreover, to determine which students are at risk of multiple risk behaviours or depressive symptoms, it was also examined if demographic characteristics could help identify at risk students. Results showed that students with a non-Dutch ethnic background reported less substance use than students of Dutch descent. This may) be due (at least partly) to their cultural and/or religious beliefs and practices related to smoking, drinking alcohol and using drugs [40]. However, students of non-Dutch descent more often reported problem behaviours compared to students with a Dutch background. Older students and students who were a parent also more often reported problem behaviours compared to their younger counterparts and to adolescents who were not a parent yet. This observation is in line with previous research showing that ethnic minority students, older students, and students who are a parent, are at increased risk of dropout [7]. Finally, girls more often reported depressive symptoms compared to boys, which is also supported by previous research [47].

The present study has some limitations. As this is a cross-sectional study, we cannot determine the direction of association between risk behaviours and depressive symptoms. While earlier research has identified depression as a predictor of risk behaviours, research has also shown that risk behaviours can be predictors of depression. Furthermore, a third factor may make youth susceptible to both depression and a wide range of behaviours [48-50]. Although our population reflects the average population in vocational schools in the Netherlands as regards age, gender, and ethnicity [29,30,39], this study was only conducted among students in the Netherlands in the two lowest levels of vocational education. Therefore, generalization to other education levels and countries should be done with caution. Moreover, almost 30% of students did not provide written consent, mainly because they were absent during the assessment and participating students for whom truancy information was not available were more likely to display risk behaviours and depressive symptoms than students for whom truancy information was available. This could have affected the generalisability of the results since non-participating students were not included in the analyses and students for whom truancy information was missing were not included when calculating prevalence of risk behaviour clusters. This limitation probably means that the prevalence of risk behaviour clusters has been underestimated. Furthermore, potential underestimation of risk behaviours clusters may have also led to underestimation of the association between risk behaviours clusters and depressive symptoms. Another limitation is the use of self-reporting for most variables included in this study, which may have resulted in less reliable outcomes. Nevertheless, research suggests that, for example, self-reported alcohol consumption among adolescents is generally valid [51].

## Conclusions

In conclusion, risk behaviours formed two separate clusters, which suggests that interventions should preferably address multiple risk behaviours simultaneously. However, more research is needed to further examine how risk behaviours cluster among adolescents and young adults and further investigations are warranted to determine if shared determinants can be identified. Furthermore, this study highlights the importance of screening for multiple risk behaviours when depressive symptoms are evident in adolescents and young adults, whereas if depressive symptoms are evident it could be useful to screen for multiple risk behaviours. Finally, the findings of this study suggest that interventions to prevent risk behaviours and depressive symptoms should target older students, girls, and students who are a parent, in particular, because these groups reported risk behaviours clusters or depressive symptoms more frequently.

## Additional files

Additional file 1:
**Associations between clusters of risk behaviours and depressive symptoms (as binary outcome) (N = 424)** ^**a**^ **.**
Additional file 2:
**Associations between demographics and depressive symptoms (as binary outcome) (N = 534)** ^**a**^ **.**
