# Supplementary material for: Depressive symptoms and clustering of risk behaviours among adolescents and young adults attending vocational education: a cross-sectional study
Source: BMC Public Health. 2015 Apr 18;15:396. doi: 10.1186/s12889-015-1692-7 (PMC4404651; doi:10.1186/s12889-015-1692-7)
Supplement: Additional file 1: — Associations between clusters of risk behaviours and depressive symptoms (as binary outcome) (N = 424) a . [file 12889_2015_1692_MOESM1_ESM.doc]

**Additional file 1. Associations between clusters of risk behaviours and depressive symptoms (as binary outcome) (N** **=** **424)^a^**

|  | **Depressive symptoms** |  |  |
| --- | --- | --- | --- |
|  | **Model 1a** | **Model1b** | **Model 2** |
|  | **OR (95% CI)** | **OR (95% CI)** | **OR (95% CI)** |
| Substance use | **1.37 (1.10 – 1.70** |  | **1.31 (1.05 – 1.63)** |
| Problem behaviours |  | **1.45 (1.13 – 1.85)** | **1.38 (1.08 – 1.77)** |

*Note:* Bold numbers indicate significant results at *P* < .05.

^a^ Logistic regression analyses.

Model 1a is adjusted for age, gender, ethnicity, being a parent and substance use.

Model 1b is adjusted for age, gender, ethnicity, being a parent and problem behaviours.

Model 2 is adjusted for age, gender, ethnicity, being a parent, substance use and problem behaviours.
