# Supplementary material for: Depressive symptoms and clustering of risk behaviours among adolescents and young adults attending vocational education: a cross-sectional study
Source: BMC Public Health. 2015 Apr 18;15:396. doi: 10.1186/s12889-015-1692-7 (PMC4404651; doi:10.1186/s12889-015-1692-7)
Supplement: Additional file 2: — Associations between demographics and depressive symptoms (as binary outcome) (N = 534) a . [file 12889_2015_1692_MOESM2_ESM.doc]

**Additional file 2.** Associations between demographics and depressive symptoms (as binary outcome) (N = 534)^a^

|  | **Depressive symptoms^b^** |
| --- | --- |
|  | **OR (95% CI)** |
| Age | **1.14 (1.05 – 1.24)** |
| Gender (ref. = boys) | **2.39 (1.56 – 3.67)** |
| Ethnicity (ref. = Dutch) | 1.15 (0.75 – 1.78) |
| Being a parent (ref. = No) | 0.56 (0.27 – 1.18) |

*Note:* Bold numbers indicate significant results at *P* < .05.

^a^ Logistic regression analyses.

^b^ Age, gender, ethnicity and being a parent are included at the same time.
